# Comparative evaluation of the algorithms for parametric mapping of the novel myocardial PET imaging agent ^18^F-FPTP

**DOI:** 10.1007/s12149-017-1171-6

**Published:** 2017-04-25

**Authors:** Ji Who Kim, Seongho Seo, Hyeon Sik Kim, Dong-Yeon Kim, Ho-Young Lee, Keon Wook Kang, Dong Soo Lee, Hee-Seung Bom, Jung-Joon Min, Jae Sung Lee

**Affiliations:** 1 Department of Nuclear Medicine, Seoul National University College of Medicine, Daehak-ro 101, Chongnogu, Seoul, 03080 Korea; 2 Department of Nuclear Medicine, Chonnam National University Hwasun Hospital, 160 Ilsimri, Hwasun, 519-809 Jeonnam Korea; 3 Department of Biomedical Sciences, Seoul National University College of Medicine, Daehak-ro 101, Chongnogu, Seoul, 03080 Korea; 4 Department of Molecular Medicine and Biopharmaceutical Sciences, Graduate School of Convergence Science and Technology, Seoul National University, 1 Gwanak-ro, Gwanak-Gu, Seoul, 08826 Korea; 5 Department of Cardiology, Chonnam National University Hwasun Hospital, 160 Ilsimri, Hwasun, 519-809 Jeonnam Korea

**Keywords:** Quantification, Parametric image, Simulation, Myocardial PET

## Abstract

**Objective:**

(^18^F-fluoropentyl)triphenylphosphonium salt (^18^F-FPTP) is a new promising myocardial PET imaging tracer. It shows high accumulation in cardiomyocytes and rapid clearance from liver. We performed compartmental analysis of ^18^F-FPTP PET images in rat and evaluated two linear analyses: linear least-squares (LLS) and a basis function method (BFM) for generating parametric images. The minimum dynamic scan duration for kinetic analysis was also investigated and computer simulation undertaken.

**Methods:**

^18^F-FPTP dynamic PET (18 min) and CT images were acquired from rats with myocardial infarction (MI) (*n* = 12). Regions of interest (ROIs) were on the left ventricle, normal myocardium, and MI region. Two-compartment (*K*_1_ and *k*_2_; 2C2P) and three-compartment (*K*_1_–*k*_3_; 3C3P) models with irreversible uptake were compared for goodness-of-fit. Partial volume and spillover correction terms (*V*_*a*_ and *α* = 1 − *V*_*a*_) were also incorporated. LLS and BFM were applied to ROI- and voxel-based kinetic parameter estimations. Results were compared with the standard ROI-based nonlinear least-squares (NLS) results of the corresponding compartment model. A simulation explored statistical properties of the estimation methods.

**Results:**

The 2C2P model was most suitable for describing ^18^F-FPTP kinetics. Average *K*_1_, *k*_2_, and *V*_*a*_ values were, respectively, 6.8 (ml/min/g), 1.1 (min^−1^), and 0.44 in normal myocardium and 1.4 (ml/min/g), 1.1 (min^−1^), and 0.32, in MI tissue. Ten minutes of data was sufficient for the estimation. LLS and BFM estimations correlated well with NLS values for the ROI level (*K*_1_: *y* = 1.06*x* + 0.13, *r* ^*2*^ = 0.96 and *y* = 1.13*x* + 0.08, *r* ^*2*^ = 0.97) and voxel level (*K*_1_: *y* = 1.22*x* − 0.30, *r* ^*2*^ = 0.90 and *y* = 1.26*x* + 0.00, *r* ^*2*^ = 0.92). Regional distribution of kinetic parametric images (*αK*_1_, *K*_1_, *k*_2_, *V*_a_) was physiologically relevant. LLS and BFM showed more robust characteristics than NLS in the simulation.

**Conclusions:**

Fast kinetics and highly specific uptake of ^18^F-FPTP by myocardium enabled quantitative analysis with the 2C2P model using only the initial 10 min of data. LLS and BFM were feasible for estimating voxel-wise parameters. These two methods will be useful for quantitative evaluation of ^18^F-FPTP distribution in myocardium and in further studies with different conditions, disease models, and species.

**Electronic supplementary material:**

The online version of this article (doi:10.1007/s12149-017-1171-6) contains supplementary material, which is available to authorized users.

## Introduction

Absolute quantification of regional myocardial blood flow (MBF) and myocardial flow reserve (MFR) on PET scans enables comprehensive evaluation of asymptomatic and symptomatic coronary artery disease (CAD) [1–3].

The most widely used tracers for myocardial PET scans are ^15^O-water, ^13^N-ammonia, and ^82^Rb which have short physical half-lives (2 min, 10 min, and 76 s, respectively) [1, 2, 4, 5]. Although ^15^O-water is a physiologically ideal myocardial perfusion agent because of its high first-pass extraction fraction and metabolically inert property, its clinical use is limited because of rapid equilibrium between myocardium and the blood pool and poor image quality [2]. ^13^N-ammonia yields markedly better image quality than ^15^O-water because ^13^N-ammonia is a soluble substance trapped in the myocardium and it has longer half-life and shorter positron range than ^15^O [1]. However, the requirement of an on-site cyclotron and synthesis module prevents wide clinical use of ^13^N-ammonia [4, 6]. In contrast, ^82^Rb does not require an on-site cyclotron and synthesis module, and its short half-life allows rapid rest and stress imaging studies for evaluating MFR. Thus, ^82^Rb is currently more widely used for clinical purposes [1, 4–6]. However, ^82^Rb PET has the limitation of relatively poor image quality because of its low extraction fraction and long positron range [1, 4, 5, 7, 8]. In addition, the high cost of monthly replacement of the ^82^Sr/^82^Rb generator limits the use of this tracer to large medical centers [6–9].

**Fig. 1:**
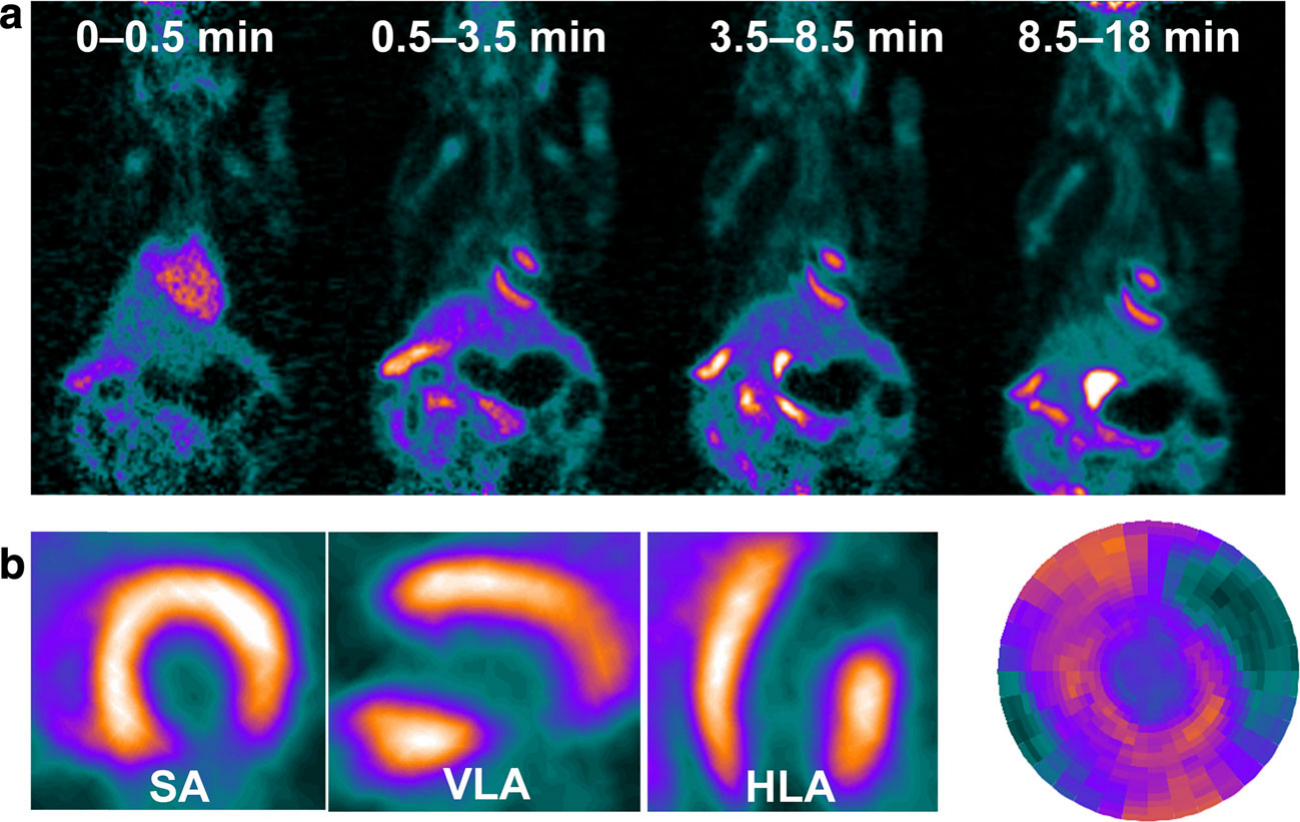
Spatiotemporal distribution of ^18^F-FPTP. **a** Serial positron emission tomography (PET) scan data from a rat with myocardial infarction showing rapid accumulation of ^18^F-FPTP in the myocardium and the marked contrast between normal myocardium and other neighboring organs such as liver and lung. **b** Short-axis, vertical long-axis, and horizontal long-axis images and a polar map of ^18^F-FPTP PET data summed between 1 and 18 min after contrast injection

The development of ^18^F-labeled myocardial PET tracers was motivated by the limitations of the existing myocardial perfusion imaging agents for PET [1, 2, 7–12]. The long half-life of ^18^F (110 min) allows more counts on the PET image, treadmill exercise for stress imaging, and delivery of labeled tracer to hospitals that do not have a cyclotron [1, 8, 9]. ^18^F also yields better PET spatial resolution than other radioisotopes because of its shorter positron range (i.e., seven times shorter than ^82^Rb) [4, 8, 9].

(^18^F-fluoropentyl)triphenylphosphonium salt (^18^F-FPTP) is a promising ^18^F-labeled myocardial imaging agent for PET, specifically accumulating to a high degree in cardiomyocytes via a negative inner transmembrane potential of mitochondria (Fig. 1; Suppl. Figure 1) [13–15]. It was also shown that ^18^F-FPTP has greater first-pass myocardial extraction than ^13^N-ammonia in isolated perfused rat heart, indicating the suitability of ^18^F-FPTP as a quantitative myocardial PET imaging agent [14, 15]. In addition, no metabolite was detected in the serum of mice 30 min after intravenous injection of ^18^F-FPTP, implying that the metabolite correction would not be necessary in the kinetic analysis of ^18^F-FPTP myocardial PET data [13]. There was also a strong correlation between myocardial defect size measured by ^18^F-FPTP PET and the hypoperfused area measured by quantitative 2,3,5-triphenyltetrazolium chloride staining in a rat myocardial infarction (MI) model [16]. Rapid washout from the liver is another merit of ^18^F-FPTP, which is advantageous during the evaluation of lateral myocardial wall uptake [2, 5, 15, 16].

In this study, we performed compartmental analysis on rat ^18^F-FPTP PET images to identify the most suitable compartment model. We then evaluated two linear analysis methods for estimating the robust kinetic parameters and generating parametric images. The minimum dynamic scan duration for kinetic analysis was also investigated. In addition, computer simulation studies were performed to confirm the two linear methods results.

## Materials and methods

### Radiochemistry

The radiotracer ^18^F-FPTP was prepared as described in a previous study [13].

### Animal model

All procedures in this study were approved by the Chonnam National University Animal Research Committee and the Guide for the Care and Use of Laboratory Animals. In this study, we used 8-week-old male Sprague–Dawley rats (*n* = 12, weight 250–260 g). To induce MI, left coronary artery occlusion surgery was performed on the rats 1 day before the PET scans.

### PET/CT procedure

Among twelve rat PET/CT data, three data acquired in our previous study [13] were included and reconstructed with our new time frame for the purposes of the current work and remaining nine data were newly acquired. In these studies, a small animal dedicated PET/CT scanner (Inveon; Siemens Medical Solutions, Knoxville, TN) was used. Dynamic PET was started with an intravenous tail-vein injection of ^18^F-FPTP and continued for 18 min (5 × 1 s, 5 × 5 s, 3 × 10 s, 4 × 15 s, 16 × 30 s, 8 × 60 s). PET images were reconstructed with 128 × 128 × 159 matrices of 0.78 × 0.78 × 0.80-mm voxel size using a filtered back-projection algorithm (Ramp filter, cutoff = 0.5). An X-ray CT transmission scan was performed after the PET scan.

### Image analysis

Regions of interest (ROIs) for the left ventricular (LV) cavity, normal myocardium, and MI region were drawn on static PET images generated by accumulating PET counts from 1 to 18 min and co-registered with the CT images of each animal using MRIcro software (http://www.mricro.com). The LV ROI to obtain the image-derived input function for kinetic analysis was drawn on the center of the LV cavity through multiple transaxial slices with the size of 6–10 voxels per slice to avoid spillover contamination from the myocardial activity. The ROI for normal myocardium was drawn on the voxels located sufficiently far from the MI and centered in the automatically drawn peak activity contour along the myocardium. The CT information was supplementary and was used for drawing ROIs on MI regions (The MI ROIs were drawn only in 10 rats because the MI was not clearly induced in 2 rats). The time-activity curves were then extracted by applying these ROIs to the serial time frames of the PET data. No metabolite correction was applied to the LV input function [13]. Time-activity curves between 0 and 10 min were used for the kinetic analysis.

Rat mode in PMOD software (version 3.6; PMOD Technologies, Zurich, Switzerland) was used to generate polar map images of ^18^F-FPTP PET. FIRE software [17] was used to reorient static PET images and fusion between parametric images.

### Compartment modeling: nonlinear analysis and model selection

**Fig. 2:**
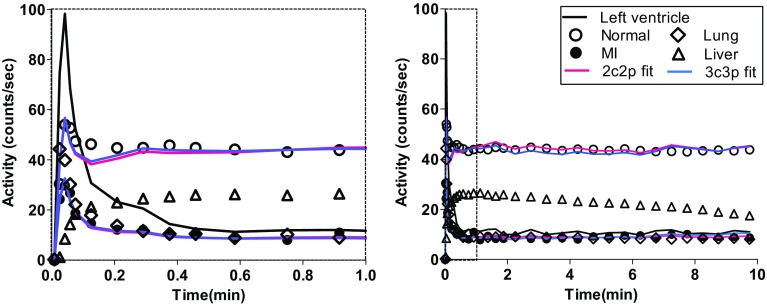
Left ventricular input function (*black line*) and tissue time-activity curves for a myocardial infarction (MI) region (*filled circle*), remote normal myocardium (*open circle*), liver (*open triangle*), and lung (*open diamond*) for two time scales (*left* 0–1 min, *right* 0–10 min). Fitting curves for the MI region and normal myocardium were obtained using two-compartment (*red line*) and three-compartment (*blue line*) models

To determine the suitable tracer kinetic model of ^18^F-FPTP PET, a two-compartment model with *K*_1_ (ml/min/g) and *k*_2_ (min^−1^) and a three-compartment model with irreversible uptake [*K*_1_, *k*_2_, and *k*_3_ (min^−1^)] (Suppl. Figure 2) were compared in terms of goodness-of-fit for the tissue time-activity curves. The three-compartment model with reversible uptake was not considered because of the obvious irreversible characteristics of this tracer, as shown in the tissue time-activity curves (Fig. 2). In each compartment model tested, a blood volume fraction (*V*_*a*_) term was incorporated in the operation equation for curve fitting [18–23]. The following equation shows how the *V*_*a*_ is incorporated into the two-compartment model equation:

1 $$C_{\text{T}} \left( t \right) = \left( {1 - V_{a} } \right)K_{1} { \exp }( - k_{2} t) \otimes C_{a} (t) + V_{a} C_{a} (t)$$

where *t* is the time, *C*_*T*_(*t*) is the tissue time-activity curve, *C*_*a*_(*t*) is the arterial input function, and *K*_1_ and *k*_2_ are, respectively, the rate constants describing the uptake to and washout from the tissue compartment.

For the model selection, the tissue time-activity curves were fitted to the models using a nonlinear least-squares (NLS) method based on the Levenberg–Marquardt algorithm [19, 24]. Akaike Information Criteria (AIC) were used to determine the most appropriate model for ^18^F-FPTP [25, 26].

### Compartment modeling: linear analysis methods and parametric image generation

In addition to the NLS curve fitting, the kinetic parameters were estimated using linear analysis methods. These methods are less vulnerable to noise and provide much faster computation. They are useful for robust estimation of kinetic parameters and generation of their parametric images [20, 23, 27, 28]. The linear analysis methods were applied only to the two-compartment model because it turned out to be more appropriate than the three-compartment model for describing the kinetics of ^18^F-FPTP (see “Results” for more detail).

We adopted two linear analysis methods. One is the multiple linear least-squares (LLS) method modified from our previous approach to the kinetic analysis of ^15^O-water [20]. The other is the basis function method (BFM), which is another well-established linear method used to generate parametric images [21, 22, 28, 29].

In addition to the ROI analysis, the LLS and BFM methods were used to generate the parametric image of each kinetic parameter through the voxel-wise parameter estimation [21, 22, 28–30] and post-Gaussian filtering with 1-mm kernel size (full width at half maximum).

### Simulation

We also performed a simulation study to explore the statistical properties of the estimation methods (NLS, LLS, BFM) tested for ^18^F-FPTP [20, 23, 27]. Average arterial input function of ^18^F-FPTP obtained from the rat studies was employed for the simulation study [20]. For generating simulated tissue time-activity curves, three levels of *K*_1_ (2.0, 6.0, and 10.0 ml/min/g) were assumed, and *V*_*a*_ and *K*_1_/*k*_2_ were fixed at 0.2 and 3.0, respectively, based on the results of rat studies. Poisson-like random noise generated with the consideration of activity and frame duration was added to the simulated tissue time-activity curves with six noise levels from 0 to 15 (voxel level time-activity curves in the rat studies had the noise level between 5 and 10) [20]. A total of 10,000 data sets were generated for each pair of *K*_1_ and noise level [20]. Finally, the coefficient of variation (CV), bias, and error in the estimation of each parameter were calculated [20, 27].

## Results

### Spatiotemporal distribution of ^18^F-FPTP

Figure 1a shows the serial PET scan data of a rat, revealing the dynamic whole-body distribution of ^18^F-FPTP. Rapid clearance of ^18^F-FPTP from blood was observed in the LV cavity region. It had rapidly accumulated in normal myocardium and was highly sustained during the whole scan duration. The high contrast between normal and MI regions in the myocardium was also well maintained until the end of the PET scan. ^18^F-FPTP uptake in the liver was quite low, making great image contrast between myocardium and liver which will be useful for mitigating the incorrect estimation of tracer uptake in the lateral wall of the heart observed with other MPI tracers, such as ^13^N-ammonia [13].

Short-axis, vertical long-axis, and horizontal long-axis images and the polar map of ^18^F-FPTP PET data summed between 1 and 18 min after injection are shown in Fig. 1b. They show excellent image contrast between myocardial uptake of ^18^F-FPTP and the LV blood pool cavity. There is clear discrimination of the MI region in the anterolateral LV wall induced by occlusion of the left coronary artery from the myocardium with intact perfusion.

Figure 2 shows the representative LV input function and tissue time-activity curves for the MI region, remote normal myocardium, liver, and lung in two time scales (0–1 and 0–10 min) in a single rat, confirming the favorable spatiotemporal properties of ^18^F-FPTP as a myocardial PET imaging agent (Fig. 1). Myocardium/blood activity ratios were 3.83 ± 0.75 (mean ± standard deviation) at 5 min, 3.73 ± 0.53 at 10 min, and 3.67 ± 0.61 at 15 min. Representative activity ratio plots in a single rat are shown in Suppl. Figure 3. They indicate that ^18^F-FPTP quickly reaches equilibrium between the myocardium and blood in rats, which is sustained until the end of the PET scan with about a four times higher ratio in normal myocardium than in the MI region.

### Nonlinear analysis and model selection

Both the two- and three-compartment model equations incorporated with *V*_*a*_ yielded an excellent NLS curve fitting as shown in Fig. 2. Kinetic parameters estimated by the NLS method using the two- and three-compartment models and their AIC values are summarized in Table 1. Both models provided much lower *K*_1_ values in the MI region relative to normal myocardium, but there were similar *k*_2_ values between the normal and MI regions. The *K*_1_, *k*_2_, and *V*_*a*_ values estimated using the two- and three-compartment models were comparable. The *k*_3_ values estimated using the three-compartment model were negligibly small, and AIC values of the two-compartment model were lower than those of three-compartment model. Accordingly, the simpler model, the two-compartment model, was selected as the optimal model for describing ^18^F-FPTP kinetics, and it was employed for subsequent linear analysis methods.

**Table 1:** Comparison of kinetic parameters estimated using two-compartment and three-compartment models and goodness-of-fit

Model	*K*_1_ (ml/min/g)	*k*_2_ (min^−1^)	*k*_3_ (min^−1^)	*V*_a_	RMSE	AIC
Normal myocardium						
2C	6.8 ± 1.8	1.1 ± 0.3	–	0.44 ± 0.10	3.07	40.8
3C	6.9 ± 1.8	1.2 ± 0.4	0.01 ± 0.01	0.43 ± 0.11	3.06	42.6
MI tissue						
2C	1.4 ± 0.5	1.1 ± 0.4	–	0.32 ± 0.11	1.19	11.3
3C	1.4 ± 0.5	1.3 ± 0.3	0.02 ± 0.02	0.33 ± 0.12	1.32	16.7

*K*_1_, *k*_2_, *k*_3_ and *V*_*a*_ data are means ± standard deviations

*RMSE* root mean square error, *AIC* Akaike information criteria, *2C* two-compartment, *3C* three-compartment

### Linear analysis and parametric image

**Fig. 3:**
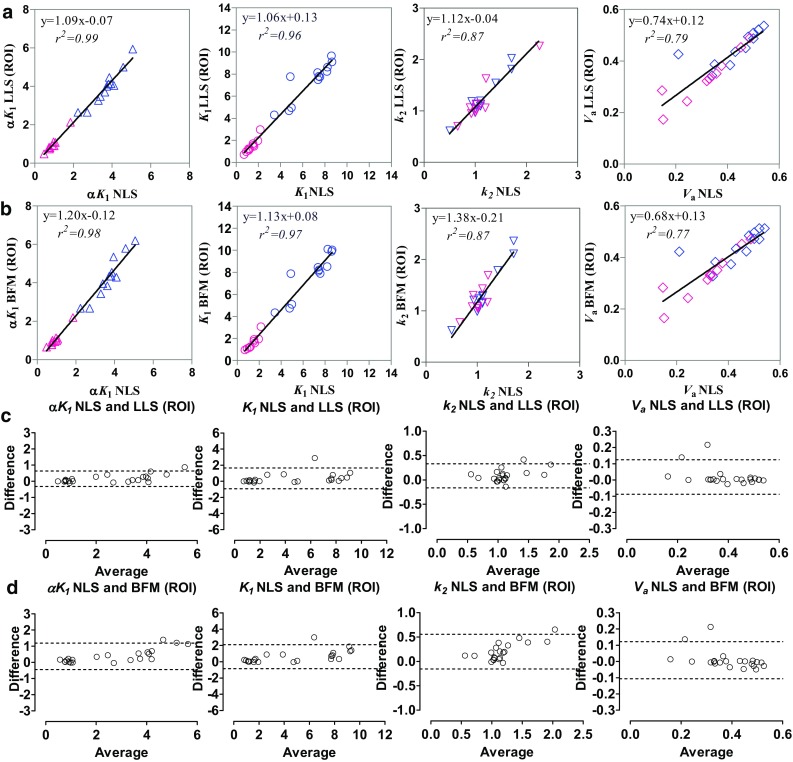
Scatter plot and Bland–Altman analysis of the kinetic parameters (*K*_1_, *k*_2_, *V*_*a*_) obtained using two linear analysis methods—linear least-squares (LLS) and basis function method (BFM) for the two-compartment model applied to the region of interest (ROIs) time-activity curves and those using nonlinear least-squares (NLS) estimates from ROI time-activity curves. **a**, **c** LLS. **b**, **d** BFM. In **a** and **b**, different colors are used for the normal (*blue*) and MI (*red*) data

The kinetic parameters obtained using the LLS and BFM methods for a two-compartment model applied to the ROI time-activity curves are correlated with the NLS estimates from ROI time-activity curves in Fig. 3. All the parameters (*K*_1_, *k*_2_, *V*_*a*_) estimated using LLS (Fig. 3a, c) and BFM (Fig. 3b, d) were well correlated with NLS estimates except for the low *V*_*a*_ values. *K*_1_ and *k*_2_ estimates showed better correlation and less deviation than the *V*_*a*_ values.

**Fig. 4:**
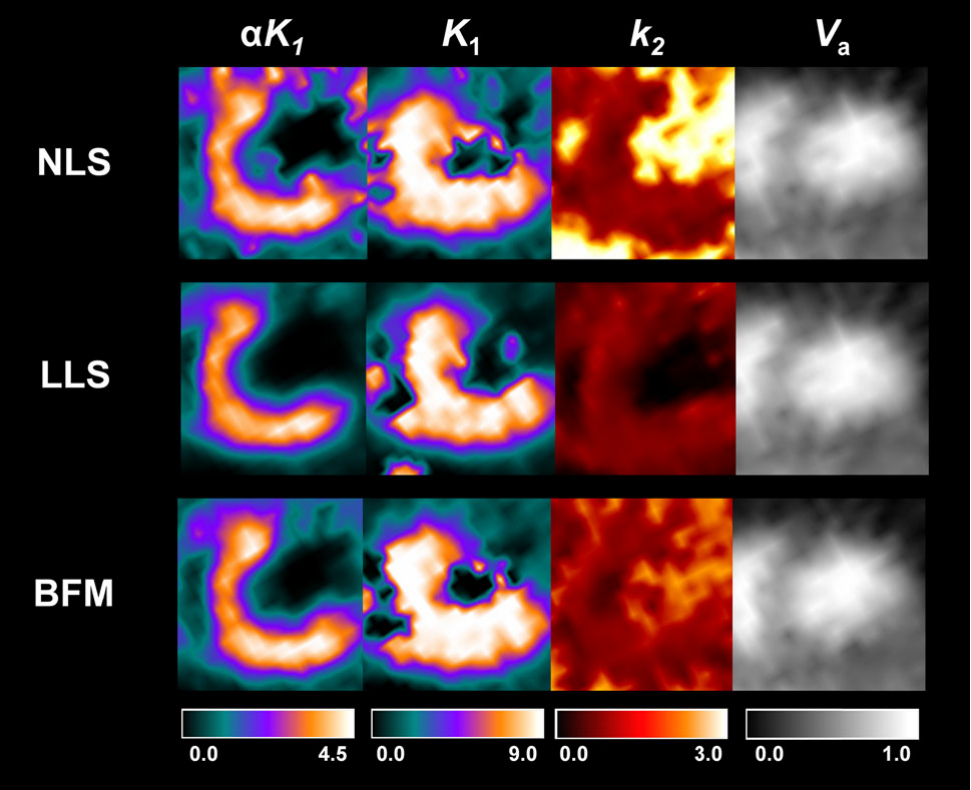
Parametric images of *αK*_1_ (*α* = 1 − *V*_*a*_), *K*_1_, *k*_2_ and *V*_*a*_ generated using NLS, LLS, and BFM (short-axis)

Figure 4 (short-axis) and Suppl. Figures 4 and 5 (vertical and horizontal long-axes) show the parametric images generated using NLS, LLS, and BFM. All three methods offered similar spatial distributions of kinetic parameters, but the two linear analysis methods (LLS and BFM) offered better image quality than NLS. The parametric images showed physiologically and physically relevant parameter distributions. The *K*_1_ images representing the partial volume and spillover corrected uptake or perfusion showed the sharp contrast between normal myocardium and the MI region and the uniform *K*_1_ distribution across the myocardium between the endocardial and epicardial walls. The *αK*_1_ (*α* = 1 − *V*_*a*_; perfusible tissue fraction or recovery coefficient [18, 19]) images offered the uptake ratio distribution weighted by partial volume effect (PVE)s. The *V*_*a*_ images illustrated well the blood pool distribution, particularly in the left and right ventricles, and spillover of blood activity into the myocardium. All three methods showed uniform *k*_2_ distribution in normal myocardium. However, the NLS and BFM methods provided too high and non-zero *k*_2_ values in both ventricles, whereas the LLS method yielded *k*_2_ parametric images with good contrast between myocardium and ventricles.

**Fig. 5:**
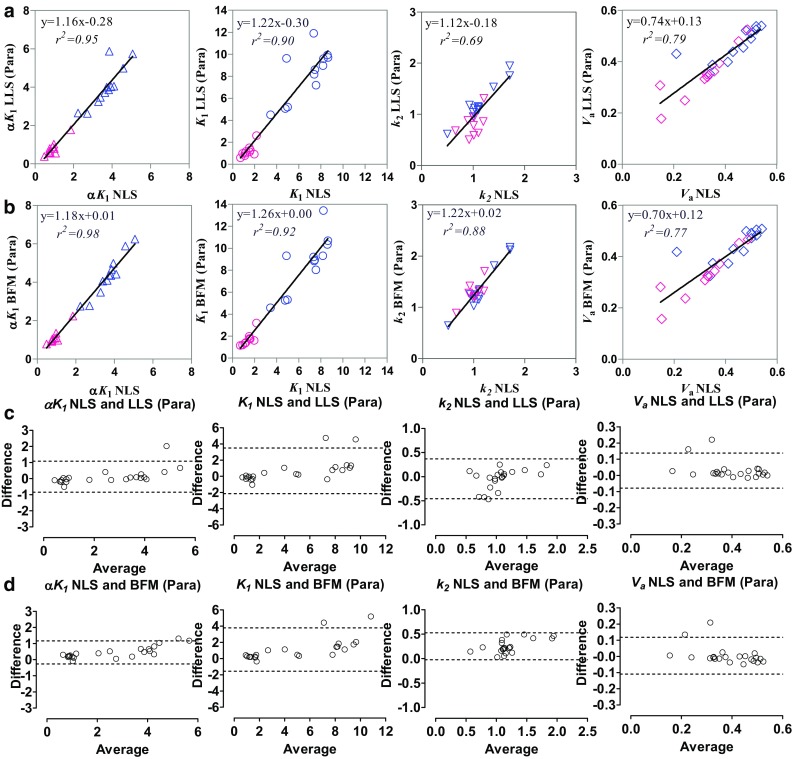
Scatter plot and Bland–Altman analysis of the kinetic parameters (*K*_1_, *k*_2_, *V*_*a*_) obtained by applying the ROIs onto the parametric images obtained using two linear analysis methods (LLS, BFM) and those using NLS estimates from ROI time-activity curves. **a**, **c**, LLS. **b**, **d**, BFM. In **a** and **b**, different colors are used for the normal (*blue*) and MI (*red*) data

Kinetic parameters were obtained by applying the ROIs to the parametric images and calculating the average parameter values within the ROIs, which were compared with the NLS estimates from ROI time-activity curves (Fig. 5a, c, LLS; Fig. 5b, d, BFM). The voxel-wise parameter estimation resulted in relatively higher *K*_1_ values, especially in the voxels with high *K*_1_.

### Scan duration

**Fig. 6:**
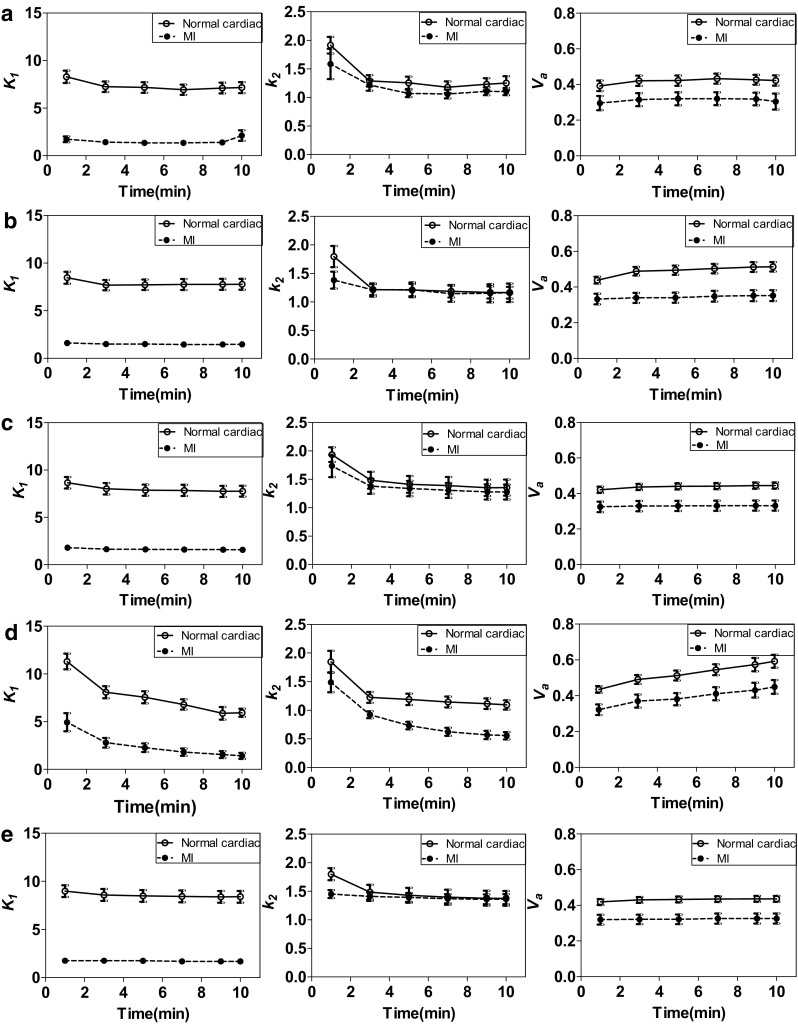
Kinetic parameters estimated with different scan durations using the **a** NLS method on ROI time-activity curves, **b** LLS method on ROI time-activity curves, **c** BFM method on ROI time-activity curves, **d** LLS parametric images, and **e** BFM parametric images

Figure 6 shows the kinetic parameters estimated using different scan durations, indicating that 5- and 10-min data collections are sufficient for estimating kinetic parameters using ROI- and voxel-wise estimations, respectively. The *K*_1_ value (the parameter of interest in MBF imaging) showed earlier convergence (even with only 3 min dynamic PET data) than other parameters in ROI analysis which is used in most clinical settings for MBF estimation.

### Simulation study

**Fig. 7:**
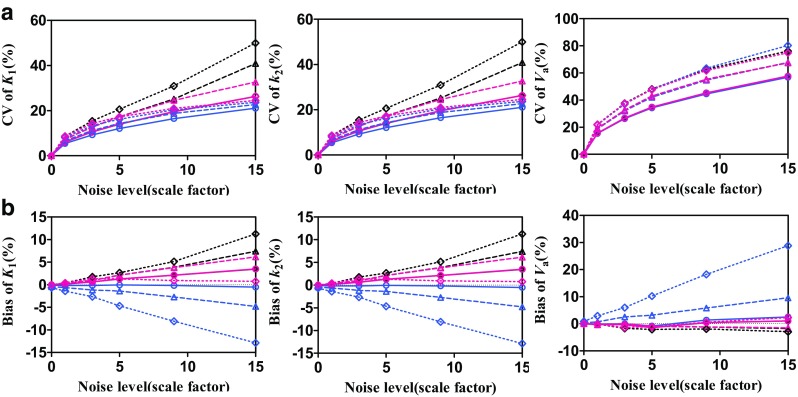
Results of the simulation study: coefficients of variation (CV) (**a**) and bias (**b**) in the estimation of each parameter using the NLS (*black symbols and lines*), LLS (*blue*), and BFM (*red*) methods. Three *K*_1_ levels were considered *open circle* 2.0, *open triangle* 6.0, *open diamond* 10.0

Figure 7 shows the CV and bias in the estimation of each parameter using the NLS, LLS, and BFM methods. The LLS and BFM showed smaller CV in parameter estimation than NLS, and LLS was superior to BFM in the estimations of *K*_1_ and *k*_2_. The BFM was the best estimation method in terms of the bias, but the LLS showed relatively higher bias in a high *K*_1_ level. Representative noisy time-activity curves for different noise levels are shown in Suppl. Figure 6.

## Discussion

In this study, we performed quantitative analysis on ^18^F-FPTP dynamic PET images from a rat model of MI to explore the spatiotemporal dynamics of this tracer, select a suitable tracer kinetic model for describing its kinetics, and investigate the properties of linear analysis methods for generating parametric images of kinetic parameters.

The model selection study for kinetic analysis suggests that the two-compartment model, which incorporates the *K*_1_, *k*_2_, and *V*_*a*_ parameters, is suitable for describing the kinetics of ^18^F-FPTP in rat myocardium. The additional tissue compartment and kinetic parameters to distinguish the free and bound tracers in cellular space could not improve the quality of curve fitting, indicating fast equilibrium between the free and bound status of ^18^F-FPTP. Easy and robust quantification of myocardial perfusion will be possible because kinetic modeling of ^18^F-FPTP does not require metabolite correction and is based on the two-compartment model.

The simplicity of two-compartment model has led to the feasibility of relatively simple linear analysis methods (LLS, BFM) for the noisy ROI kinetic analysis and parametric image generation. In the simulation study, the LLS and BFM showed the lowest variation and bias, respectively (Fig. 7), in agreement with the indications shown in the parametric images generated based on them (Fig. 4). The high correlation between the parameters obtained using LLS and BFM methods and those from the NLS ROI analysis, which is the gold standard but requires a long computation time, has also verified the feasibility of the linear analysis methods (Figs. 5, 7). The parametric images of ^18^F-FPTP provided physiologically relevant results (Fig. 4; Suppl. Figures 4 and 5). The *K*_1_ and *αK*_1_ parametric images (flow-related images with and without partial volume correction) well accounted for the perfusion difference between normal myocardium and MI regions. Although the *K*_1_ image provided partial volume-corrected perfusion information, the image quality was worse than that with *αK*_1_, mainly because of the division operation required for obtaining *K*_1_ values. The distribution of *k*_2_ was more uniform than *K*_1_ or *αK*_1_, and *V*_*a*_ images clearly showed the blood pool distribution in the heart. However, only the LLS showed good contrast between myocardium and the LV cavity in *k*_2_ parametric images, which may be caused by the better parameter estimation performance of lower parameter values than attained with the other methods.

In this study, input function was obtained from LV pool of PET images. Although the kinetic model itself includes PVE, the input function is assumed to be free from the PVE as in our previous rat PET study [31]. However, the PVE on the input function is not avoidable sometimes even in the small animal dedicated PET scanners. Previously we have shown that LV input function in mice PET studies is underestimated by 5–10% relative to the arterial blood samples [32]. However, in this study, we used more advanced small animal PET scanner than the previous study (Inveon vs. Focus 120) [33–35]. In addition, rats have much larger LV cavity than mice, causing less severe PVE. In case there is the underestimation of LV input function due to the PVE, it would more influence on the *K*_1_ and *V*_*a*_ (overestimation) rather than *k*_2_.

Usually, a weighting factor is considered for compensating the different signal-to-noise ratios (SNRs) among PET time frames because the different SNRs cause bias in parameter estimation. However, the weighting did not prove effective in this study for improving the bias properties and parametric image quality (Suppl. Figure 7; frame duration was used as the weighting factor). The weight would be more effective for the radiotracers with binding and dissociation as parameters of interest and the relatively short half-life of the radioisotope [27]. However, it seems that the slope of the early frames with rapid buildup and the ratio of tissue and blood activity in the later frames are the most dominant factors when determining the kinetic parameters of ^18^F-FPTP. The parameters determined mainly in each of these early and later portions of the time-activity curves are influenced very little by the weighting factors.

## Conclusion

A novel, promising, myocardial PET imaging agent, ^18^F-FPTP, was quantitatively evaluated in rats with MI. For describing the kinetics of this tracer, a two-compartment model was more suitable than a three-compartment model. Two linear analyses for parametric image generation showed that the kinetic parameters were well correlated with nonlinearly estimated gold standard ones and yielded the high quality images of those parameters. The first 10 min of the dynamic scans was sufficient for obtaining accurate kinetic parameters in quantitative PET studies. Finally, the two algorithms for parametric mapping of ^18^F-FPTP in myocardium will be useful for further studies under different conditions, disease model, and species.

### Electronic supplementary material

Below is the link to the electronic supplementary material.

Supplementary material 1 (DOCX 1286 kb)

## Appendix

In the LLS method, the measured arterial input function and tissue time-activity curve are related to the ideal ones, as shown in the following equations, by incorporating partial-volume and spillover effects [9, 12, 18–23]:

2 $$\tilde{C}_{a} \left( t \right) = C_{a} (t)$$

3 $$\tilde{C}_{T} \left( t \right) = \left( {1 - V_{a} } \right)C_{T} \left( t \right) + V_{a} C_{a} (t)$$

The ideal input function and tissue time-activity curve expressed with the measured curves are then obtained by transposing the above equations and substituting them into the differential equation for an ideal tissue time-activity curve (d*C*_*T*_/d*t* = *K*_1_ *C*_*a*_ − *k*_2_ *C*_*T*_), yielding the following equation [20]:

4 $$\frac{\text{d}}{{{\text{d}}t}}\left\{ {\tilde{C}_{T} \left( t \right) - V_{a} \tilde{C}_{a} \left( t \right)} \right\} = \left( {1 - V_{a} } \right)K_{1} \tilde{C}_{a} \left( t \right) - k_{2} \left\{ {\tilde{C}_{T} \left( t \right) - V_{a} \tilde{C}_{a} \left( t \right)} \right\}.$$

By rearranging this equation and applying integration to both sides, the following linear equation in the form of ***y*** = ***Xθ*** is derived:

5 $$\begin{aligned} \varvec{y} & = \left[ {\tilde{C}_{T} \left( t \right)} \right] \hfill \\ \varvec{X} & = \left[ {\tilde{C}_{a} \left( t \right)} {\space}{\space}{\space} {\mathop \int \nolimits \tilde{C}_{a} \left( t \right){\text{d}}t} {\space}{\space}{\space} {\mathop \int \nolimits \tilde{C}_{T} \left( t \right){\text{d}}t} \right] \hfill \\\varvec{\theta} & = \left[ {V_{a} } {\space} {\space} {\space} {\left( {1 - V_{a} } \right)K_{1} + k_{2} V_{a} }{\space}{\space}{\space} { - k_{2} } \right]^{\varvec{T}} \end{aligned}$$

Then, an estimated ***θ*** based on LLS criteria was given by the ***θ*** = (***X*** ^*T*^ ***X***)^−1^ ***X*** ^*T*^ ***y***, and the LLS solution of (*K*_1_, *k*_2_, *V*_*a*_) is analytically calculated from the ***θ***.

For applying BFM, a set of linear models was derived from Eq. 1 as follows:

6 $$C_{\text{T}} \left( t \right) = \left( {1 - V_{a} } \right)K_{1} B_{j} \left( t \right) + V_{a} C_{a} \left( t \right) \; {\text{for}} \; 1 \le j \le m$$

where each *B*_*j*_(*t*) = exp(*k*_2,*j*_·*t*) ⨂*C*_*a*_(*t*) is a basis function, *k*_2,*j*_ is the *j*th value in a predefined discrete pool of *k*_2_ values, and *m* is the number of the predefined *k*_2_ values. We assumed that *k*_2_ is bounded between 0.01 and 2.5 (min^−1^) based on the estimated *k*_2_ values using the NLS method. Then, 100 basis functions with discrete *k*_2_ values spaced logarithmically between the bounds were generated, and an LLS estimate of (*K*_1_, *V*_*a*_) was obtained for each basis function. Finally, the BFM solution of (*K*_1_, *k*_2_, *V*_*a*_) was determined as the one with the smallest sum of squared errors between measured and estimated tissue time-activity curves.

## Abbreviations

MBF: Myocardial blood flow
MFR: Myocardial flow reserve
^18^F-FPTP: (^18^F-fluoropentyl)triphenylphosphonium salt
CAD: Coronary artery disease
MI: Myocardial infarction
LV: Left ventricular
ROI: Regions of interest
*C*_a_: Tracer arterial blood radioactivity concentration
*C*_T_: Activity concentration in tissue concentration
*K*_1_,*k*_2_,*k*_3_: Rate constant
*V*_*a*_: Blood volume fraction
*Α*: Perfusible tissue fraction or recovery coefficient; 1 − *V*_*a*_
AIC: Akaike information criterion
NLS: Nonlinear least-squares method
LLS: Linear least-squares method
BFM: Basis function method
CV: Coefficient of variation
