# Supplementary material for: Comparative evaluation of the algorithms for parametric mapping of the novel myocardial PET imaging agent 18F-FPTP
Source: Ann Nucl Med. 2017 Apr 25;31(6):469–79. doi: 10.1007/s12149-017-1171-6 (PMC5486518; doi:10.1007/s12149-017-1171-6)
Supplement: Supplementary file 1 — Supplementary material 1 (DOCX 1286 kb) [file 12149_2017_1171_MOESM1_ESM.docx]

**Suppl. Fig. 1** Chemical structure of ^18^F-FPTP.

**Suppl. Fig. 2** Two candidate models for describing the kinetics of ^18^F-FPTP in rat myocardium. (a) Two-compartment model. (b) Three-compartment model.

**Suppl. Fig. 3** Myocardium/blood activity ratios (○: normal myocardium, ●: myocardial infarction).

**Suppl. Fig. 4** Parametric images of *αK*_1_ (*α* = 1 − *V_a_*), *K*_1_, *k*_2_, and *V_a_* generated using NLS, LLS, and BFM (vertical long-axis).

**Suppl. Fig. 5** Parametric images of *αK*_1_ (*α* = 1 − *V_a_*), *K*_1_, *k*_2_, and *V_a_* generated using NLS, LLS, and BFM (horizontal long-axis).

**Suppl. Fig. 6** Representative noisy time−activity curves for different noise levels used in the simulation study.

**Suppl. Fig. 7** The effect of weighting on parametric image generation.
